# Deleterious Results from Wearing Artificial Teeth Set upon the Continuous Rubber Base

**Published:** 1875-07

**Authors:** J. C. C. Downing


### ARTICLE V.

### *Deleterious Results from Wearing Artificial Teeth Set Upon the Continuous Rubber Base.*

BY DR. J. C. C. DOWNING.

Case 1.—Mrs. B. consulted me May 7th, 1874, native of United States, æt. 43, married second time; states that she has been sick, or feeling miserable, for over six months; has been under treatment of another regular physician during that time. Head symptoms: occasional dizziness and confusion of ideas, and sluggishness of the memory; restlessness, and inability to obtain refreshing sleep at night; depression of spirits. Eyes: dimness of vision, muscæe volitantes, photophobia, increased lachrymation; pain, deep seated; from the iritis present, could not obtain a satisfactory ophthalmoscopic examination. Nasal secretions altering in excess, or deficient. Mucous lining of mouth, throat, tongue, gums, etc., congested, and irregularly covered with various sized patches of superficial ulceration. Painful deglutition, increased salivary secretions. Fetor of breath. Skin of the arms, neck, chest, and to a greater or less extent, the rest of the body, covered with an eruption in various stages of progress, attended with a sense of heat, dryness, and itching. Pains in the bones which are worse at night. Loss of appetite. Irregular menstruation.

As she was wearing artificial teeth at the time of my visit (on account of the soreness of the gums) I did not have my attention drawn to them. Improvement under treatment was slow, with frequent relapses. After three months had elapsed I discovered that, whenever the state of the mouth would allow of it, she wore the articles mentioned above. I examined them, and discovered that the polish was gone, and under a small lens the surface appeared studded with minute excavations, like needle pricks. She had worn them sixteen years, and was incredulous when I asserted that I held in my hands the whole cause of her sickness. Why had they never troubled her before? She consented, however, to wear them only when needed to masticate food, and I directed her husband to apply to them frequently, a shellac varnish. From that time the case was in every way, steadily improved.

Case 2.—Mrs. C., æt. 59; has been in good and improving health. Recently had a change made in artificial teeth worn; now has a set as above. Requested me to examine her mouth. Discovered the mucous lining of lower gums and cheeks congested, tender, and showing several small, superficial, white, eroded, painful spots. Stated it as my opinion, that the mercury used in the manufacture of the plate was being slowly decomposed, from its union to other substances, and was acting both locally, and probably constitutionally. Moreover, on examing the set, I noticed a plug of amalgam in the concave side of the plate, which the dentist had said, was inserted to fill a cavity caused by an air bubble during its manufacture. Mrs. C. then told that her old family physician had given her a similar opinion, of the correctness of which she is now convinced. I would like to ask, are such cases of frequent occurrence? May not such causes be overlooked, if the local effects are not marked, but the general or constitutional ones are?—*Medical and Surgical Reporter.*

---

